# Development of a new measure to check attitude towards water conservation^✰^

**DOI:** 10.1016/j.mex.2022.101992

**Published:** 2023-01-20

**Authors:** R. Archana Reddy, Rakesh Sengupta, B. Micheal Jackson, Christelle Lewis

**Affiliations:** SR University, Warangal, India 506371

**Keywords:** Water conservation, Attitudes, Attitude scale, Perceived water right, Moral obligation to save water, Development of a new measure to check attitude towards water conservation

## Abstract

Water Conservation is the need of the hour and attitudes towards conserving the resources is what is sure to become a priority in the coming years. In order to understand what could influence the change in attitude and thereby bring about a change of behavior, we first need to shift our attention from understanding the water crisis to understanding the existing attitude of the society towards a water crisis. In the current work we address the current attitude towards water conservation by providing baseline data about Indians’ attitudes and behavior/behavioral intentions in conserving water. We introduce a scale constructed to test the Attitude towards water conservation in India. The scale consists of 20 items divided into 5 sub-scales. We conducted a nationwide survey on 430 Participants and their responses were checked for reliability. The internal consistency values of all 5 scales were ranged between 0.68 and 0.73

• Out of the 15 questions from Attitude towards water conservation from Dolnicar, S., & Hurlimann, A. (2010) 1 question was changed to suit Indian context
• 5 questions were added to show Perceived Moral Obligation, Behavioral Intensions and Perceived Water Rights

## Graphical abstract

**Figure:**
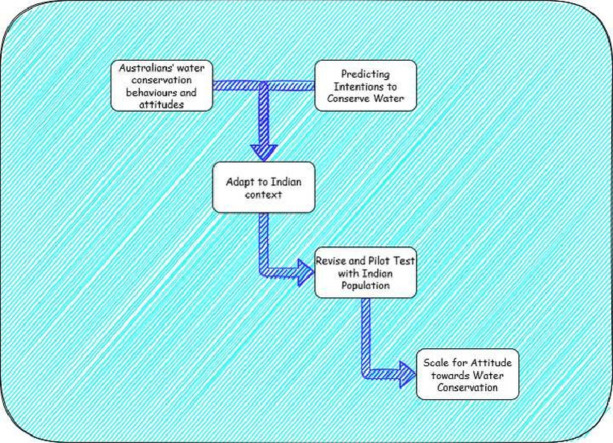

Specifications table

**Table:** 

Subject area:	Psychology
More specific subject area:	*Behavioral psychology*
Name of your method:	*Development of a new measure to check attitude towards water conservation*
Name and reference of original method:	1. The General Attitude towards water conservation (GAWC): The 11 items under this factor tapped awareness of people towards water scarcity and their perceived scope towards the increasing need to conserve water. The questions were adopted from Australians' Water Conservation behaviors and Attitudes [15]. Perceived Moral Obligation (PMO): Behavior could often be influenced by moral ethics. This could further extend to why we engage in certain social acts. The items of PMO measures the extent to which one feels obligated to conserve water, Lam, S. P. (1999) Behavioral Intentions (BI): The items in this factor measure the extent to which one ‘attitude reflects in their actions. Lam [1] 7. Perceived Water Rights (PWR): Depending on one's opinion on what their right towards the natural resources is, their attitude towards its depletion could vary. Lam SP (1999) measures the extent to which people think of access to water as their basic right.
Resource availability:	*Yes.* *The data can be found in the following link* *https://drive.google.com/drive/folders/1401lMip6rekQ1S2OQx5rGkaJlbm59sd0?usp=sharing* *We analyzed the data using SPSS software on a windows 10 laptop (i3- 7th gen, 8 Gb ram)*

## Method details

### Questionnaire construction

We made a database on previous water conservation and the scales and questions that were not relevant to the purpose of the study were filtered out. Some questions were improvised so as to fit the purpose of the study as well as the choice of sample. Finally, a 20-item questionnaire with 5 sub-scales was constructed to check the attitude towards water conservation in India.

### Sub-Scales

1. The General Attitude towards water conservation (GAWC): The 11 items under this factor tapped awareness of people towards water scarcity and their perceived scope towards the increasing need to conserve water. The questions were adopted from Australians' Water Conservation Behaviors and Attitudes [15]. Their questions were derived from insights based on a qualitative study they previously did. The items in this sub-scale included questions or statements about the general attitude towards water conservation, for example, \More attention to water conservation is needed.”, \Water Storage issues don't affect me.”, \The need for water conservation depends on location.” and so forth.
2. Past Experience and Behavior (PE): The 3 items in this sub-scale emphasize the participant's prior encounter with water shortage and the efforts they have/have not put into conserving water; i.e., variable factors that may or may not affect the attitude to conserve water. For example: \I conserve water wherever I can.”, \ I advocate water conservation.”, etc.
3. Perceived Moral Obligation (PMO): Behavior could often be influenced by moral ethics. This could further extend to why we engage in certain social acts. The items of PMO measures the extent to which one feels obligated to conserve water. For instance, the 2 items are \Water is a natural resource, everybody is obligated to treasure it.”, \Everybody should save water because water resources are limited.”
4. Behavioral Intentions (BI): The items in this factor measure the extent to which one's attitude reflects in their action. The questions being, \If circumstances allowed you, would you like to reduce water consumption at home?”, \If circumstances allowed you, would you like to change or install some water-saving appliances?”
5. Perceived Water Rights (PWR): Depending on one's opinion on what their right towards the natural resources are, their attitude towards its depletion could vary. PWR Lam [1] measures the extent to which people think of access to water as their basic right. For example \Everybody has the right to use water according to his/her own interest, and the government should satisfy everyone's demand.” Nuanced Analysis of Water Resource (NAWR): The item in this sub-scale is an ambiguous question; a subtle portal that would tell us if the person's attitude towards water conservation is limited to just conserving water or if they think that other factors affect the Water problems we face in India. for example, afforestation, Reusing water, etc. The statement states that conserving water alone can save India's water problem. If the participant strongly agrees to this, then the attitude towards water conservation is only superficial. The Sub-scale NAWR should be seen alongside the GAWC sub-scale to interpret results. Other one-item scales were included for qualitative purposes. The questions across the sub-scales are listed in Table 1.

**Table 1:** Attitude Scale for Water Conservation containing 5 primary Sub-Scales i.e., The General Attitude towards water conservation (GAWC), Past Behavior (PE), Perceived Moral Obligation (PMO), Behavioral Intentions (BI), and Nuanced analysis of water resources (NAWR) as well as the 2 secondary sub-scales Perceived Water Rights (PWR) and Past Experience (PE).

Sub- Scale	Item no.	Question/Statement
GAWC	1	More attention to water conservation is needed
	3	I am very positive about water conservation
	4	I could make more effort to conserve water
	8*	I feel no pressure to conserve water at the moment
	9*	Water Shortage issues don't affect me.
	10*	I am not concerned at all with water conservation
	11*	Water conservation isn't my responsibility.
	12*	It is challenge to convince others to conserve water
	13*	The need for water conservation depends on location
	19	Water conservation is important
	20	Water conservation is necessary because of water scarcity
PB	2	I conserve water whenever I can
	5	I advocate water conservation among my friends and family
PE	6	I have experienced limited water supply before.
NAWR	7	Water conservation alone can solve India's water problem
PMO	14	Water is a natural resource; everybody is obliged to treasure it
	15	Everybody should save water because water resources are limited
PWR	16	Everybody has the right to use water according to his/her own interest, and the government should satisfy everyone's demand.
BI	17	If circumstances allowed you, would you like to reduce water consumption at home?
	18	If circumstances allowed you, would you like to change or install some water-saving appliances?

Items marked with an asterisk (*) were negatively scored (revered scoring), these questions are worded negatively.

### Scoring

A 5-Point Likert Scale was used to collect responses for each question. The scale ranged from Strongly Agree to Strongly Disagree. For the complete questionnaire, see Supplementary Material .The items had been segregated for positive scoring (Items: 1,2,3,4,5,6,7,14,15,16,17,18,19,20) and negative scoring (Items: 8,9,10,11,12,13). For Positively scored questions, Strongly agree received a score of 5, Agree received a score of 4, and so on. For Negatively scored questions, the scoring was reversed; Strongly agree received a score of 1, agree received a score of 2, and so on. The scores were carefully selected so as to attribute high scores to a positive attitude towards water conservation. For example, in Question 1, Strongly agreeing to the statement \More attention to water conservation is needed” indicates a positive attitude towards water conservation hence Strongly agree is scores as 5. Whereas, in Question 10, Strongly agreeing to the statement \I am not concerned at all with water conservation" does not indicate a positive attitude towards water conservation. Therefore, for a negatively scored statement, a reverse scoring is observed where strongly agree is given a score of 1, agree is given a score of 2, and so forth.

### Range of scores

For positive and negative scoring (after reverse scoring), a maximum score is obtained by the choice that scored 5 for all the questions/items whereas, a minimum score is obtained by the choice of 1 for all the questions/ items. The sub-scale GAWC/ General attitude towards water conservation has 11 items Score ranges from a maximum of 55 to a minimum of 5. The sub-scale PBI/ Past Behavior or Experience has 3 items therefore the maximum score an individual can obtain is 15 and the minimum score is 3. The sub-scale PMO/ Perceived moral obligation to conserve water has two items therefore the maximum and minimum score an individual can obtain from this sub-scale is 5 and 2 respectively. The sub-scale PWR/ Perceived Water-Right has only a single question thus the range of score is between 5 and 1. Sub-scale BI/ Behavioral Intentions has 2 items therefore the maximum and minimum score will be 10 and 2 respectively. 5 primary sub-scales (GAWC, PB, PMO, BI, NAWR) are added to obtain the total score of the attitude scale. The maximum score an individual can score is 100 and the minimum is 20.

### Data collection

We collected 450 responses from participants all over the country. We rejected 20 responses due to incomplete data. Survey Monkey -a data collection app & website, was used to collect participants' responses on the questionnaire in the English Language. Weblinks, QR codes, and Social Networking templates helped the participants access the survey used to collect responses. We analyzed the collected responses.

## Results

In order to assess the reliability, or internal consistency, of the given set of scale or test items we used Cronbach's Alpha. Select questions from the questionnaire were grouped under the Past Experience factor considering that the nature of the questions was not inclined towards capturing the change in attitude but carried information important enough to contribute towards a change. The value for General Attitude towards water conservation falls in acceptable range with 0.705. Behavioral Intentions sub-scale carries a greater and acceptable value of 0.734. The Sub-scale for a perceived moral obligation has 2 items with an internal consistency of 0.685. We analyzed the reliability of sub-scales that contained more than one item. Table 2 shows the results for the internal consistency of three sub-scales i.e., General Attitude towards water conservation (GAWC), Perceived Moral Obligation to Conserve Water (PMO), and Behavioral Intentions (BI) analyzed by Cronbach's alpha. GAWC has an internal consistency with a Guttman's split-half reliability of 0. 701 (Table 3). There was an overall above-average positive attitude towards water conservation with a mean score of 79.03 (SD = 8.56) out of a total of 100 which amounts to a 79.03% inclination rate per participant.

**Table 2:** Mean (M), Standard Deviation (SD), and Reliability; Chronbach's α and Split-half reliability (Guttman's split-half coefficient), of Primary Sub-scales: General Attitude towards water conservation (GAWC), Perceived Moral Obligation to Conserve Water (PMO), and Behavioural Intentions (BI). Here, N denotes the number of items analyzed. The first half of GAWC consists of: Item1,3,4 8,9, & 10 & its second half consists of Item 11,12,13,19,20.

Sub-scale	N	M	SD	Reliability					
				Cronbach's *α*	Split-Half Reliability				
					Gullman's COF	Cronbach's *α*			
						1sthalf		2ndhalf	
						*α*	N	*α*	N
GAWC	11	41.51	5.614	.705	.701	0.60	6	.464	5
PMO	2	8.95	1.207	.685	–	–	–	–	–
BI	2	8.56	1.286	.734	–	–	–	–	–

**Table 3:** Pearson's pairwise correlation of sub-scales GAWC, PMO, PWR, and BI.

Pearson's correlation	GAWC	PMO	BI	PWR
GAWC	1			
PMO	0.258 *P* < 0.01	1		
BI	0.224 *P* < 0.01	.451 *P* < 0.01	1	
PWR	0.484 *P* < 0.01	⁎⁎	⁎⁎	1

⁎⁎ No significant relation observed.

**Table 4:** Pearson's pairwise correlation of sub-scales GAWC, PMO, PWR, and BI with sub-scale PB/E (Item2: I conserve water whenever possible. Item5: I advocate water conservation among friends and family Item6: I have experienced limited water supply before) and NAWR (Water conservation alone can solve India's water problem).

	Item2	Item5	Item6	NAWR
GAWC	0.219⁎⁎*P* = 0.00	0.386⁎⁎*P* = 0.00	0.257⁎⁎*P* = 0.00	0.368⁎⁎*P* = 0.00
PMO	0.209⁎⁎*P* = 0.00	0.260⁎⁎*P* = 0.00	0.139⁎⁎*P* = 0.00	0.162⁎⁎*P* = 0.01
PWR	0.039 *P* = 0.445	0.059 *P* = 0.18	0.087 *P* = 0.068	0.171⁎⁎*P* = 0.00
BI	0.244⁎⁎*P* = 0.00	0.345⁎⁎*P* = 0.00	0.178⁎⁎*P* = 0.00	0.165⁎⁎*P* = 0.01

⁎⁎ Significance at level 0.01(2 tailed).

**Table 5:** Pearson's pairwise correlation between PB/ E (Item 2,5 & 6) and NAWR.

	Item2	Item5	Item6	NAWR
Item2	1			
Item5	0.371⁎⁎*P* = 0.00	1		
Item6	0.243⁎⁎*P* = 0.00	0.222⁎⁎*P* = 0.00	1	
NAWR	0.282⁎⁎*P* = 0.00	0.322⁎⁎*P* = 0.00	0.191⁎⁎*P* = 0.00	1

⁎⁎ Significance at level 0.01(2 tailed).

(Note: Cronbach's alpha above 0.9 is considered Excellent, Value between 0.9 and 0.8 is Good, Value between 0.8 and 0.7 is acceptable, Value between 0.7 and 0.6 is Questionable, Value between 0.6 and 0.5 is Poor, and Values below 0.5 is considered Unacceptable.

Since the Cronbach's alpha Value was just below Acceptable range, we performed Guttman's split-half reliability because Cronbach's alpha tends to under-estimate the true reliability. Guttman's split-half reliability is better suited since the Sample size is adequate and the number of items is small)

**Fig. 1:**
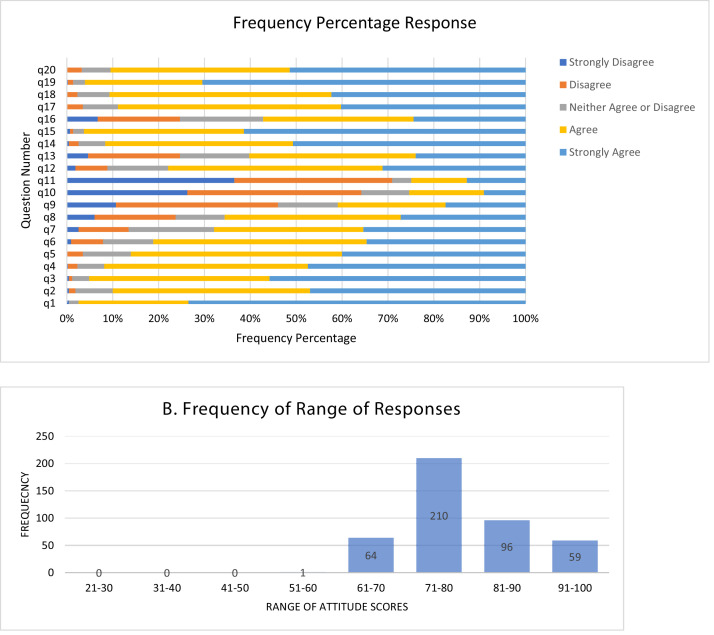
A: Frequency Percentage of Responses for all items in the Attitude Towards Water Conservation Scale (ATWC). This data is inclusive of the primary and the secondary sub-scales The legends represent the scores (after reverse scoring) given for the responses. Figure 1B: Frequency percentage of the range of responses of the entire sample. Minimum and the maximum score that can be obtained is 20 and 100 respectively.

Fig. 1B illustrates a frequency percentage of the samples' attitudes towards water conservation. These scores are of that concerning only the primary sub-scales. The frequency of responses is shown in Table A1. Each question received different responses from different participants. Fig. 1 shows the frequency percentage of responses for each attitude choice against each question. Please check Tables A1 and A2 for the same. Table A2 shows the frequency percentage of responses that were derived based on data shown in Table A1. (See Supplementary Material for Tables and figures).

In order access the relationship between the sub-scales Pearson's pairwise correlation was conducted; results of which indicate that there was a significant positive association between General attitude towards water conservation and Perceived Moral obligation to save water. (r (430) =0.258, *p* < 0.01). The general attitude towards water conservation was correlated with the Perceived Water Rights (r (430) =0.482, *P* < 0.01). There is no correlation between Perceived water rights and Perceived moral obligation to conserve water; Nor is any correlation observed between Perceived water rights and Behavioral Intention. A significant positive association is observed between Behavioral Intention and Perceived moral obligation (r (430) =0.451, *P* < 0.01). A significant positive association was also observed between General attitude towards water conservation and Behavioral intentions to conserve water (r (430) =0.224, *P* < 0.01).

(Note: Pearson's correlation Coefficient values can range from +1 to −1, where +1 indicates a perfect positive relationship, −1 indicates a perfect negative relationship, and a 0 indicates no relationship exists.

If the value is near ± 1, then it said to be a perfect correlation: as one variable increases, the other variable tends to also increase (if positive) or decrease (if negative))

Results of Pearson's 2 tailed pairwise correlation indicates that there was a significant positive association between the conservation of water (Item 2) and advocating others to conserve water (Item 5) (r (430) =0.371, *P* < 0.01), conservation of water (Item 2) and experiences of limited water supply in the past (Item 6) (r (430) =0.243, *P* < 0.01), advocating water conservation (Item 5) and experiences of limited water supply in the past (Item 6) (r (430) =0.222, *P* < 0.01). Pearson's pairwise correlation of Item 2,5, and 6 all showed a negative association with the statement on Nuanced Analysis of Water Resource. I.e., There is no negative association with the belief that Water conservation alone can solve India's water problems (NAWR statement) with practice in conserving water (Item 2) (r (430) =−0.282, *P* < 0.01), advocated water conservation (Item 5) (r (430) =−0.371, *P* < 0.01) and experiences with limited water supply in the past (r (430) =−0.191, *P* < 0.01)

## Introduction

Over the next few years, numerous river basins across the world are likely to experience water scarcity [2]. Per capita annual water availability in India was about 1545 cubic m in 2011, which has been predicted to fall to 1486 due to rise in population from 1.2 billion to 1.3 billion (Press release from Ministry of JAL SHAKTI India on 25 March 2021). The Falkenmark Index is a commonly used measure of water scarcity and a country with per capita annual renewable water below 1700 m^3^ is said to be under water stress. Annual precipitation in India has been estimated at about 4000 bm^3^(Billion Cubic Meter) and sources potential is 1869bm^3^. Due to topographical and other constraints, the utilizable water resource potential is 690bm^3^ of surface water and 447bm^3^ of groundwater, totaling to 1137bm^3^. In addition to these constraints water availability in India also has huge variations with respect to location, resulting in surplus water in some river basins/regions and water scarcity in others, frequently at the same time [3].

Note: (The Falkenmark Water Stress Index measures water scarcity as the amount of renewable freshwater that is available for each person each year. A country is said to be experiencing water stress when water availability is below 1,700 m3 per person per year; below 1,000 m3 is considered water scarcity; and below 500 m3 is absolute or severe water scarcity)

Groundwater, the primary source of fresh water in the world is being used at a faster rate than it gets replenished [4]. The scarcity is a result of not just the demand for limited water resources, but also the flawed planning, management, economic policies, and other social factors. The failure to plan and conserve a natural resource is due to the lack of awareness of the need to conserve the resource or the public's lack of acknowledging the moral obligation to conserve water [2]. Compared to other natural phenomena like meteorological effects, the withdrawal of groundwater due to urbanization; especially in cities, is the major factor in the depletion of groundwater resources [5]. Various cities in India currently suffer from water shortages. Most middle-class residents of Delhi- the richest city in India, do not have dependable access to clean water sources. India has been warned by the World Bank to be on the brink of a severe water crisis. The reports indicate that the groundwater is disappearing and the rivers are turning into make-shift sewers. The situation has been too grave for just the Central Ground Water Authority [6] to regulate groundwater development. Since aquifers have no boundaries akin to political boundaries, a state's (and a country's) inappropriate management of its resources is to have a severe impact on the neighboring states and countries [7]. This may cause more political problems between states and/or countries. Despite the gravity of the situation, there is no immediate action being taken to make water conservation a priority; this is because people are still doing the same thing they did thousands of years ago, their consumption of water resources has only multiplied due to the adoption of the modernistic lifestyle. An important component of water management is to reduce the demand through water conservation Postel (1985). The only remedy to avert the crisis is to create new technologies for reusing water and encouraging people to conserve water. For example, the government can compute a water budget specific to lakes [8] and rivers etc and access quality of ground water sources used for irrigation [9] and other activities can also help to understand the water scarcity problem.

positive A attitude towards water conservation could be the first step in what might be a long journey ahead to solve India's water crisis. Although the leap is small, it's a good place to start. At the rate of how much India is modernizing, an individual stance to conserve water in a day-to-day life will only be good on the conscience but also make people responsible citizens. Awareness about water conservation is therefore important for more people to support the active implementation of water conservation. Various studies have been conducted by water authorities across India, government departments, and independent and academic research work. Despite these studies, little is known about India's attitude towards conservation and engaging in the same, particularly over the past decade. In order to understand what could influence the change in attitude and thereby bring about a change of behavior, we shift our attention from understanding the water crisis to understanding the existing attitude of the society towards a water crisis. Ajzen's theory of planned behavior [10] proposes that intentions are the cognitive means to direct our beliefs and perception towards an intent to act, and further towards taking action. Intentions are further deemed to be influenced by our attitudes and, our attitudes change amongst many others with the subjective norm (to engage in a behavior because we see it as a socially-expected behavior) and perceived behavioral control (to perceive the behavior as something that we can engage in [11]. We address the current attitude towards water conservation by providing baseline data about Indians’ attitudes and behavior/behavioral intentions in conserving water. The data further aided in the development and standardizing of a tool to quantify the attitude towards water conservation. Since the tool is derived from Ajzen's theory of Planned Behavior, it would help predict the attitude towards water conservation.

The Attitude scale for water conservation construes 5 primary sub-scales, namely:

1. Attitude towards water conservation
2. Past Behavior
3. Moral Obligation to save water resources
4. Behavioral intention of conserving water resources
5. Perceived water right

The purpose of the study is to understand and construct an attitude scale in acknowledgment of the fact that attitudes influence behavioral intentions as well as behavior and therefore, represents a crucial antecedent construct that agents of persuasion target. It also consists of two other questions from two scales that act as grouping variables or moderators of attitude.

The two secondary sub-scales are:

1. Past Experience
2. Nuanced Analysis of Water Resources

## Discussion

It has been previously established that the attitudes and beliefs of consumers directly impact water use behaviors which are closely linked to water demand [12]. To assess the relationship between general and specific environmental beliefs and their effects on pro-environmental behavior, an attitude scale was developed. Results seem to indicate that for most people the water conservation scales are reliable and valid. Reliability was assessed by Cronbach's alpha, and split-half method as well as comparing the reliability scored to scores of the that of the original scale from which the questions were derived from producing adequate values in every case.

The 20-item questionnaire has 5 sub-scales. The reliability of the 5 sub-scales was analyzed for all 20 questions answered by 430 participants. The Cronbach's coefficient assessing the internal consistency of the Scale General Attitude towards water conservation (GAWC) is 0.684 and the Guttman's’ split-half reliability of 0.738; both these coefficient scores attest to a considerably good reliability scale in the Indian population. The reason for an average score could be due to an anchoring effect caused by varied experiences with domestic water usage and societal pressures different people across different states in India experience. The study shows that people who have a moral obligation to conserve water, exhibit a positive attitude towards water conservation that reflects further into a behavioral intention to conserve water resources. The limited supply/ shortage of water conservation they may have experienced in the past could have instilled a positive attitude towards water conservation as well as a moral obligation to conserve water. Experiencing a limited water supply has also led to a behavioral intention to conserve water. People who do have a strong positive attitude towards water conservation, perceive a moral obligation to conserve water, or who have experienced limited water supply in the past, do not believe that water conservation alone can solve India's water problems. Either they are aware of other the circumstances that cause water shortage or they are responsible citizens with high moral conduct that sees conservation of water as relative to other problems. People who strongly believe that they have the right to use water would feel less obligated to save water. Hence, we had expected the moral obligation to be negatively correlated to the perceived water right. However, we found no such results from our sample. People who have a generally positive attitude towards water conservation believe that they are entitled to water resources. These results are contradictory to the majority of results about the nuanced analysis of water conservation where there is a negative correlation between the belief that water conservation alone can solve India's water problems, and advocating water conservation among others, a behavioral intention, and a perceived moral obligation to conserve water. Thus, in totality, a nuanced analysis could tell us more about how positive peoples’ attitudes towards water conservation. There is also a positive correlation between experiences of water shortage in the past and perceived water rights. This could only tell us that their water shortage situation was perceived as an unjust reason that caused their water shortage; political or otherwise.

The scale is suitable to tap the attitude towards water conservation by excluding Sub-Scale PE/Past Experience (experience of water shortage in the past), for it could vary from person to person and place to place. It could better serve as a grouping variable instead. The sub-scale PWR/ Perceived water right had ambiguous correlations that need to be modeled. Thus, it could act as a moderator instead of an independent variable. Exclusion of the score from this scale should also be observed when considering the attitude score.

## Conclusion

Water- a natural resource is considered as being bountiful. In real-time, the general public finds it hard to acknowledge that they are in the 11th hour before a severe water crisis. Rawl's, in his Text ‘A Theory of Justice’ [13] argues that capitalist institutions must define and protect primary goods such as natural resources as higher-order goods. It is the very foundation stone that all other of man's deliberate needs are satisfied, whatever that may be. Primary goods are of the highest interest or need for what a rational man requires. Their existence revolves around it although it attributes no market price onto itself; yet. With more of the primary goods being available, success is assured in carrying out man's intentions and advancements, whatsoever it may be. Water is one such higher-order good, the value of which people need to acknowledge. A society that is primarily concerned with useful information can neglect to account for the importance of these goods even when it's on the verge of a crisis.

To fix a problem, it is important to know how severe it is. Knowing the extent to which people are aware of the problem provides the foremost step; For, something as important as water conservation needs a collecting effort. Determining people's motives towards saving water is important in designing educational urban water-saving strategies [14], hence understanding attitude and behavior towards consumption towards the resource is vital to its conservation.

Based on this study we can see that majority of the people show an above-average positive attitude towards water conservation that can be tapped with the standardized Attitude scale for water conservation that contains 5 sub-scales all of which have reliability within the range of .68 and .73 reliability coefficient. There are positive associations between the general attitude towards water conservation, perceived moral obligation to conserve water, and a behavioral intention of the same. Thus, these variables determine the quality of the attitude. The NAWR should be considered alongside the GASWC, PMO, & BI to determine the depth of their analysis. The sum of the five primary sub-scales (NAWR, GASWC, PMO, BI & PB) would determine each individual's attitude score. The Sub-scale PWR and PE (Secondary sub-scales) can be included along with the questionnaire for qualitative purposes.

In this study we did not categorize based on age, gender and geographical conditions. Future studies should incorporate these Categories in order to find an even robust measure to gauge the attitude Indian Population.

## Ethics statements

This work described has been carried out in accordance with The Code of Ethics of the World Medical Association (Declaration of Helsinki) for experiments involving humans; EU Directive 2010/63/EU for animal experiments; Uniform Requirements for manuscripts submitted to Biomedical journals. Informed consent was obtained for experimentation with human subjects. The privacy rights of human subjects have been observed.

## Declaration of Competing Interest

Please **tick** the appropriate statement below (please do not delete either statement) and declare any financial interests/personal relationships which may affect your work in the box below.

The authors declare that they have no known competing financial interests or personal relationships that could have appeared to influence the work reported in this paper.

The authors declare the following financial interests/personal relationships which may be considered as potential competing interests: Please declare any financial interests/personal relationships which may be considered as potential competing interests here.

## Figures and Tables

**Table A1:** Response frequency-the frequency of the five choices against each question was estimated after reverse scoring of negatively scored questions. The astric beside the questions denote those questions which were scored in reverse.

Q no.	Response frequency				
	1	2	3	4	5
1	2	0	9	103	316
2	2	6	35	185	202
3	2	3	16	169	240
4	1	9	25	191	204
5	0	15	45	198	172
6	4	30	47	200	149
7*	11	47	80	140	152
8*	26	76	46	165	117
9*	46	152	56	101	75
10*	113	163	45	70	39
11*	157	148	18	52	55
12*	8	30	57	201	134
13*	20	86	65	156	103
14	2	9	25	176	218
15	3	3	10	150	264
16	29	77	78	141	105
17	0	15	33	209	173
18	0	10	30	208	182
19	1	5	11	110	303
20	0	14	27	168	221

**Table A2:** Frequency percentage of responses based on data from Table A1.

	Frequency percentage of responses (%)				
	1	2	3	4	5
	0.47	0.00	2.09	23.95	73.49
	0.47	1.40	8.14	43.02	46.98
	0.47	0.70	3.72	39.30	55.81
	0.23	2.09	5.81	44.42	47.44
	0.00	3.49	10.47	46.05	40.00
	0.93	6.98	10.93	46.51	34.65
	2.56	10.93	18.60	32.56	35.35
	6.05	17.67	10.70	38.37	27.21
	10.70	35.35	13.02	23.49	17.44
	26.28	37.91	10.47	16.28	9.07
	36.51	34.42	4.19	12.09	12.79
	1.86	6.98	13.26	46.74	31.16
	4.65	20.00	15.12	36.28	23.95
	0.47	2.09	5.81	40.93	50.70
	0.70	0.70	2.33	34.88	61.40
	6.74	17.91	18.14	32.79	24.42
	0.00	3.49	7.67	48.60	40.23
	0.00	2.33	6.98	48.37	42.33
	0.23	1.16	2.56	25.58	70.47
	0.00	3.26	6.28	39.07	51.40
Average	4.97	10.44	8.81	35.97	39.81

## Data Availability

I have shared the link to the data in the Submited Article
